# Stronger associations of the phase angle than the TyG index with micro- and macrovascular complications in patients with type 2 diabetes

**DOI:** 10.1186/s12944-025-02534-5

**Published:** 2025-04-01

**Authors:** Ling Liu, Yunqiang He, Yan Wang, Juming Tao, Jiachen Wang, Fangzhou Lu, Qi Fu, Tao Yang, Jingyang Gao, Shuai Zheng

**Affiliations:** Department of Endocrinology and Metabolism, The First Affiliated Hospital of Nanjing Medical University, #300 Guangzhou Road, Nanjing, 210029 China

**Keywords:** Microalbuminuria, Brachial-ankle pulse wave velocity, Phase angle, Triglyceride glucose index, Type 2 diabetes

## Abstract

**Background:**

Identifying micro- and macrovascular damage through microalbuminuria and arterial stiffness is essential for preventing renal and cardiovascular complications in patients with type 2 diabetes mellitus (T2D). The primary goal of this research is to investigate the association of the phase angle (PA), triglyceride‒glucose (TyG) index, and homeostasis model assessment for insulin resistance (HOMA-IR) with microalbuminuria and arterial stiffness in patients with T2D.

**Methods:**

In this retrospective cross-sectional study, 938 participants with T2D were enrolled. The PA was calculated from bioelectrical impedance analysis. Logistic regression was used to analyze the association of PA, the TyG index and HOMA-IR with microalbuminuria (urinary albumin-to-creatinine ratio [UACR] > 30 mg/g using overnight urine) and increased arterial stiffness (brachial-ankle pulse wave velocity [baPWV] > 1400 cm/s), respectively. Potential nonlinear relationships between PA, the TyG index, and the prevalence of microalbuminuria and increased arterial stiffness were assessed via restricted cubic splines (RCS). Subgroup analysis evaluated the robustness of the association.

**Results:**

PA was inversely correlated with the UACR (*r* = -0.29, *P* < 0.001) and baPWV (*r* = -0.37, *P* < 0.001). Confounder-adjusted analyses revealed that the highest tertile of PA was significantly associated with lower prevalences of both microalbuminuria and increased arterial stiffness than the lowest tertile, with ORs of 0.305 and 0.467 and *P* trends < 0.001 and 0.017, respectively. Conversely, the highest TyG tertile was associated with increased prevalences of microalbuminuria and increased arterial stiffness, with ORs of 1.727 and 1.625, respectively, but the *P* trends were not statistically significant. There were no significant associations between HOMA-IR and microalbuminuria and increased arterial stiffness. RCS analysis further confirmed a significant linear relationship between PA and both vascular complications. Subgroup analyses consistently demonstrated the association between PA and microalbuminuria across all subgroups stratified by sex, age, BMI, HbA1c, and duration of diabetes (all *P* < 0.01).

**Conclusions:**

Compared with the TyG index and HOMA-IR, PA is independently and more strongly associated with microalbuminuria and increased arterial stiffness in patients with T2D.

**Supplementary Information:**

The online version contains supplementary material available at 10.1186/s12944-025-02534-5.

## Introduction

Diabetic vascular complications, including both microangiopathy and macroangiopathy, represent some of the most common comorbidities in diabetes patients. These complications are significantly associated with an increased risk of morbidity and mortality [1]. Early identification of vascular damage through markers such as microalbuminuria or arterial stiffness is crucial for preventing cardiovascular and renal complications in patients with diabetes mellitus [2]. However, this method of evaluating vascular complications is both complex and expensive. Therefore, identifying new approaches to tackle or prevent the development of these complications among patients with diabetes as early as possible is essential [3].

Bioelectrical impedance analysis (BIA) is a noninvasive method that is the most widely used technique in body composition analysis, primarily to assess muscle mass and hydration [4]. One of the key parameters derived from BIA, the phase angle (PA), is calculated directly from reactance and resistance [5]. The PA represents the integrity of the cell membrane and fluid distribution between the intracellular and extracellular compartments [5]. Higher PA values ​​signify better cell integrity and function, reflecting healthier and more resilient cells. In contrast, lower PA values ​​may indicate impaired cell health or the presence of malnutrition [6]. PA has demonstrated its prognostic utility in various chronic conditions, such as obesity, cancer, liver cirrhosis, and heart failure [7–10]. Several studies have shown that patients with type 2 diabetes (T2D) generally present lower PA values than healthy individuals [11–13]. Since PA is associated with inflammation, oxidative stress, and cellular damage, PA is linked to these disease states [4, 14]. However, the role of PA in diabetic patients, particularly concerning vascular complications, remains underexplored, which may be proposed as a promising tool for simplifying and enhancing the evaluation process.

Insulin resistance (IR) is considered an essential risk factor for vascular damage and cardiovascular disease (CVD) [15, 16]. The homeostasis model assessment for insulin resistance (HOMA-IR) and triglyceride-glucose (TyG) index, which is derived from fasting blood glucose (FPG) and triglyceride (TG) levels, are both suggested as surrogate markers for IR. However, recent studies have shown that the TyG index is superior to the HOMA-IR in predicting arterial stiffness and CVD [17, 18]. Furthermore, an elevated TyG index was significantly associated with a higher risk of arterial stiffness and nephric microvascular damage in diabetic and community‑dwelling elderly individuals [19, 20].

The aim of this study was to investigate the associations of PA, the TyG index, and HOMA-IR with microalbuminuria and arterial stiffness via brachial-ankle pulse wave velocity (baPWV) in subjects with T2D.

## Methods

### Study design and participants

**Fig. 1:**
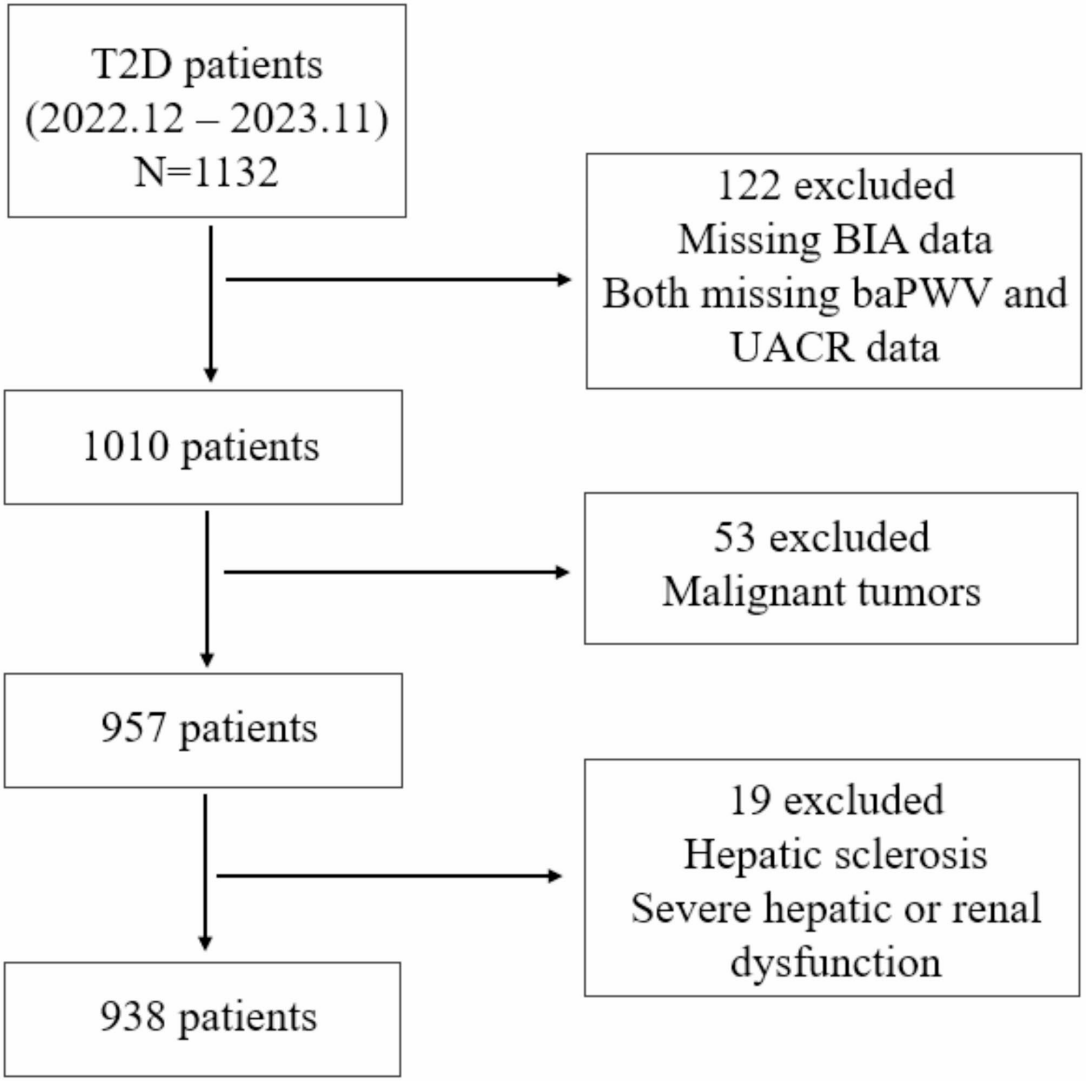
Flowchart of participant selection. Abbreviations: T2D, type 2 diabetes; BIA, bioelectric impedance analysis; baPWV, brachial-ankle pulse wave velocity; UACR, urinary albumin‒creatinine ratio

This was a retrospective cross-sectional study in the real world. A total of 938 inpatients diagnosed with T2D were enrolled from the Department of Endocrinology and Metabolism at the First Affiliated Hospital of Nanjing Medical University between December 2022 and November 2023. The process of patient selection is illustrated in Fig. 1. Criteria for inclusion in the T2D group included a previous diagnosis of T2D according to the American Diabetes Association (ADA) criteria [21]. The exclusion criteria were as follows: (1) under the age of 18; (2) the presence of malignant tumors; (3) hepatic sclerosis (fibrosis-4 score [FIB-4]>2.67; FIB-4 was calculated by the following formula: FIB-4 = Age (years) × AST (U/L) / [platelet (10^9^/L) × ALT^1/2^ (U/L)]) [22] or severe hepatic (ALT or AST ≥ 3-fold of the upper limit of the normal reference) or renal dysfunction (serum creatinine ≥ 176.8 µmol/L); and (4) incomplete laboratory examinations or BIA data. The study was approved by the First Affiliated Hospital of Nanjing Medical University Institutional Review Board (2021-SR-298).

### Data collection

Baseline characteristics were obtained from electronic hospital records. Demographic information and medical history, including sex, age, duration of diabetes, smoking status, alcohol consumption, history of hypertension, and coronary heart disease (CHD), were extracted. Anthropometric assessments involved weight, height, waist circumference (WC), systolic blood pressure (SBP), and diastolic blood pressure (DBP). Body mass index (BMI) was calculated as weight (kg)/height squared (m^2^).

Blood and urine samples were collected following an overnight fasting period for all participants. The laboratory test indicators included FPG, fasting serum insulin (FINS), glycosylated hemoglobin (HbA1c), TG, low-density lipoprotein cholesterol (LDL-C), high-density lipoprotein cholesterol (HDL-C), total cholesterol (TC), uric acid, serum creatinine, and the urinary albumin-to-creatinine ratio (UACR). Microalbuminuria was defined as a UACR over 30 mg/g. The 2-hour postprandial blood glucose (2 h-PG) levels were measured following a 75 g oral glucose tolerance test. HOMA-IR was calculated as follows: FPG (mmol/L) × FINS (mIU/L)/22.5 [23]. The TyG index was calculated as ln[TG (mg/dL) × FPG (mg/dL)/2] [24]. The estimated glomerular filtration rate (eGFR) (ml/min/1.73 m^2^) was calculated via the chronic kidney disease (CKD) Epidemiology Collaboration (CKD-EPI) equation [25].

### Measurement of baPWV and body composition parameters

The automatic measurement of baPWV was performed with the Omron Colin BP-203RPE III device (Omron Health Care, Kyoto, Japan). We adopted the average value of right and left baPWV for analysis after the participants rested for at least 5 min. A baPWV > 1400 cm/s indicates increased arterial stiffness [26]. PA was determined by using a BIA analyzer (Seca-mBCA 515, Hamburg, Germany). The abdominal visceral fat area (VFA) and subcutaneous fat area (SFA) were estimated via the Omron DUALSCAN BIA machine (Omron HDS-2000, Kyoto, Japan). The participants were required to remove metal items before performing the BIA.

### Statistical analysis

The data are presented as the means ± SDs, numbers (%), or medians (interquartile ranges). We applied Student’s t-test or Mann–Whitney U test for continuous variables and the χ2 test for categorical variables. Spearman correlations between PA and the other variables were calculated and are presented as a heatmap. Logistic regression models were used to determine the independent effects of PA, the TyG index, and HOMA-IR on microalbuminuria and increased arterial stiffness. Adjustments were performed for Model 1: age and sex; Model 2: age, sex, BMI, duration of diabetes, SBP, DBP, HbA1c, LDL, HDL, eGFR, hypertension, coronary heart disease, smoking, and drinking. Potential nonlinear relationships between PA, the TyG index, and the prevalence of microalbuminuria and increased arterial stiffness were assessed via restricted cubic splines (RCS). Additionally, we explored the association between PA, the TyG index, and the prevalence of microalbuminuria and increased arterial stiffness in subgroup analysis, stratified by sex, age, BMI, HbA1c, and duration of diabetes, with complete adjustments in Model 2.

Statistical analyses were conducted using SPSS 26.0 and R software (version 4.2.1). A two-sided *P* < 0.05 was considered statistically significant.

## Results

### Baseline characteristics

Data from 938 subjects with T2D were included in the current analysis. As illustrated in Table 1, the mean age of the study participants was 55.59 (SD, 13.84) years, and 581 were men (61.9%). The mean PA was 4.81 (SD, 0.70), and the mean TyG index was 9.64 (SD, 0.70). The prevalence rates of microalbuminuria and increased arterial stiffness were 24.5% (229/935) and 69.3% (650/938), respectively. Men exhibited higher levels of WC, DBP, uric acid, PA, and VFA, but lower levels of age, SBP, FPG, 2 h-PG, LDL-C, HDL-C, TC, HOMA-IR, eGFR, UACR, baPWV, the TyG index, and SFA compared to women (all *P* < 0.05). Additionally, men had higher rates of current smoking, drinking, and shorter diabetes duration (< 5 years) than women (all *P* < 0.05).

**Table 1:** Baseline characteristics of the T2D patients

	Overall (*n* = 938)	Male (*n* = 581)	Female (*n* = 357)	*P* value
Age (years)	55.59 (13.84)	54.04 (13.93)	58.12 (13.34)	< 0.001
Duration of diabetes				0.008
< 5 years	348 (37.1)	235 (40.4)	113 (31.7)	
≥ 5 years	590 (62.9)	346 (59.6)	244 (68.3)	
Hypertention, *n* (%)	432 (46.1)	256 (44.1)	176 (49.3)	0.121
CHD, *n* (%)	113 (12.0)	74 (12.7)	39 (10.9)	0.470
BMI, kg/m^2^	24.51 (22.39, 26.75)	24.64 (22.65, 26.89)	24.26 (22.01, 26.40)	0.146
WC, cm	91.32 (10.00)	92.62 (9.77)	89.21 (10.01)	< 0.001
SBP, mmHg	129.04 (18.06)	128.00 (17.82)	130.75 (18.34)	0.023
DBP, mmHg	77.52 (11.56)	78.80 (11.73)	75.44 (10.97)	< 0.001
FPG, mmol/L	6.80 (1.82)	6.57 (1.83)	7.17 (1.75)	< 0.001
2 h-PG, mmol/L	16.95 (3.62)	16.52 (3.53)	17.64 (3.65)	< 0.001
HbA1c, %	8.87 (2.04)	8.89 (2.09)	8.84 (1.95)	0.717
TG, mmol/L	1.36 (0.94, 2.03)	1.34 (0.92, 2.06)	1.43 (1.03, 1.97)	0.142
LDL-C, mmol/L	2.84 (2.25, 3.45)	2.78 (2.23, 3.29)	2.98 (2.29, 3.65)	0.001
HDL-C, mmol/L	1.07 (0.92, 1.26)	1.03 (0.89, 1.15)	1.18 (0.99, 1.36)	< 0.001
TC, mmol/L	4.52 (3.76, 5.28)	4.39 (3.64, 5.07)	4.76 (3.89, 5.65)	< 0.001
HOMA-IR	1.48 (0.78, 2.81)	1.24 (0.68, 2.37)	1.94 (1.10, 3.31)	< 0.001
Uric acid, µmol/L	321.90 (92.64)	338.04 (93.12)	295.59 (85.67)	< 0.001
eGFR (mL/min per 1.73 m^2^)	99.77 (19.70)	98.64 (19.66)	101.61 (19.66)	0.025
UACR, mg/mmol	1.08 (0.58, 2.92)	0.92 (0.50, 2.66)	1.24 (0.80, 3.36)	< 0.001
BaPWV, cm/s	1607.18 (333.94)	1578.50 (327.81)	1653.85 (338.99)	0.001
PA, °	4.81 (0.70)	5.02 (0.66)	4.46 (0.63)	< 0.001
TyG index	9.64 (0.70)	9.59 (0.70)	9.72 (0.68)	0.004
VFA, cm^2^	80.00 (56.20, 104.90)	83.10 (56.10, 110.90)	77.20 (56.30, 97.43)	0.012
SFA, cm^2^	176.00 (138.90, 219.20)	172.40 (138.05, 212.55)	184.05 (141.68, 235.50)	0.012
Current smoking, *n* (%)	254 (27.1)	249 (42.9)	5 (1.4)	< 0.001
Current drinking, *n* (%)	251 (26.8)	243 (41.8)	8 (2.2)	< 0.001

The data are presented as mean ± SD, numbers (%), or medians (interquartile ranges)

Abbreviations: CHD, coronary heart disease; BMI, body mass index; WC, waist circumference; SBP, systolic blood pressure; DBP, diastolic blood pressure; FPG, fasting plasma glucose; 2 h-PG, 2-hour postprandial blood glucose; HbA1c, glycated hemoglobin; TG, triglycerides; LDL-C, low-density lipoprotein cholesterol; HDL-C, high-density lipoprotein cholesterol; TC, total cholesterol; HOMA-IR, homeostasis model assessment for insulin resistance; eGFR, estimated glomerular filtration rate; UACR, urinary albumin‒creatinine ratio; BaPWV, brachial-ankle pulse wave velocity; PA, phase angle; TyG, triglyceride‒glucose index; VFA, visceral fat area; SFA, subcutaneous fat area

### Spearman correlations between PA and various clinical parameters

**Fig. 2:**
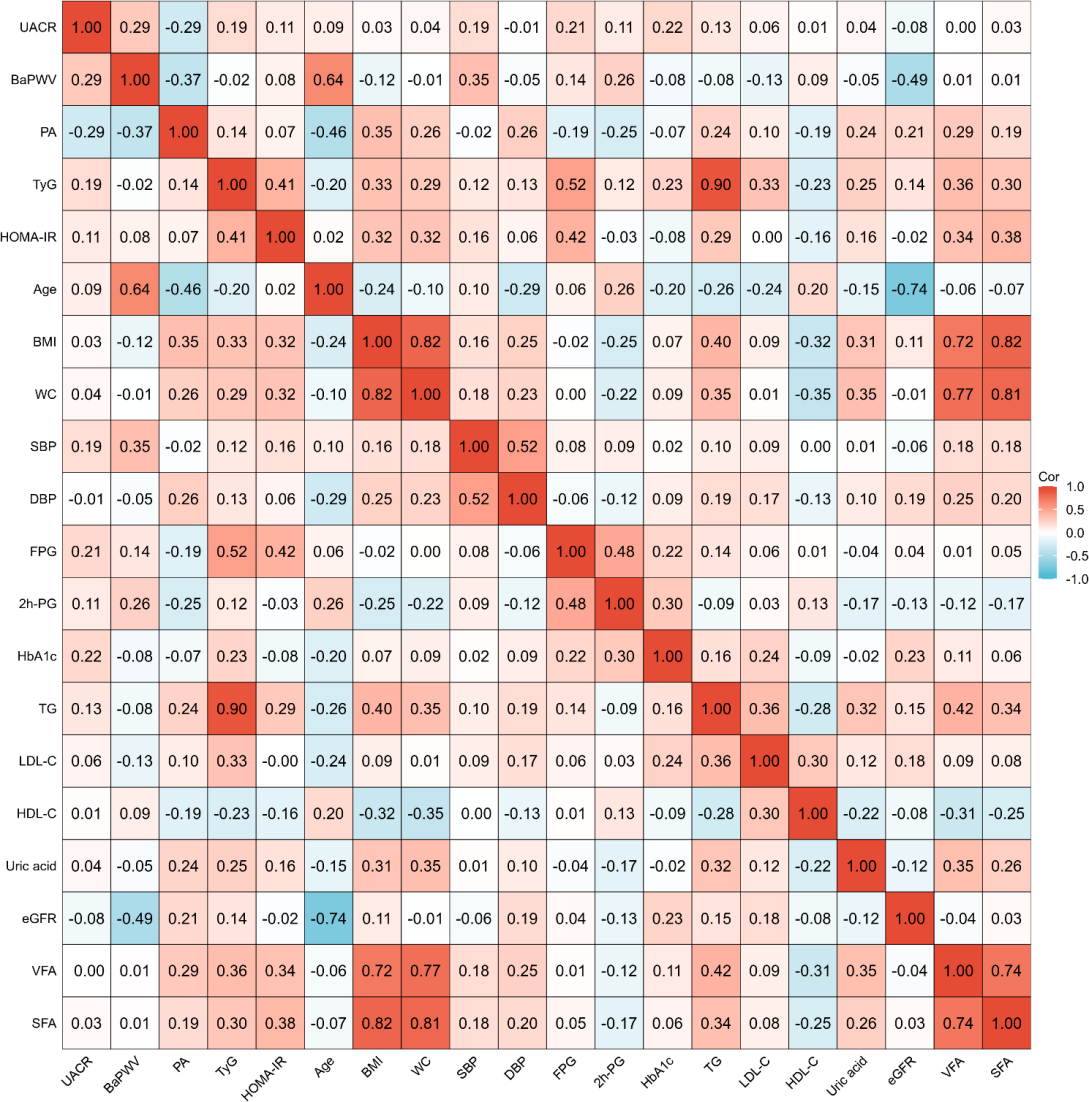
The heatmap depicts the relationship between the phase angle and the other variables. Abbreviations: UACR, urinary albumin‒creatinine ratio; BaPWV, brachial‒ankle pulse wave velocity; PA, phase angle; TyG, triglyceride‒glucose index; HOMA‒IR, homeostasis model assessment for insulin resistance; BMI, body mass index; WC, waist circumference; SBP, systolic blood pressure; DBP, diastolic blood pressure; FPG, fasting plasma glucose; 2 h-PG, 2-hour postprandial blood glucose; HbA1c, glycated hemoglobin; TG, triglycerides; LDL‒C, low-density lipoprotein cholesterol; HDL‒C, high-density lipoprotein cholesterol; eGFR, estimated glomerular filtration rate; VFA, visceral fat area; SFA, subcutaneous fat area

As shown in the heatmap in Fig. 2, PA was significantly negatively correlated with UACR (*r* = -0.29, *P* < 0.001) and baPWV (*r* = -0.37, *P* < 0.001). Additionally, PA was inversely correlated with age, FPG, 2 h-PG, and HDL-C (all *P* < 0.001) and exhibited a weak correlation with HbA1c (*P* < 0.05). Conversely, PA was positively correlated with BMI, WC, DBP, TG, uric acid, eGFR, VFA, SFA, and the TyG index (all *P* < 0.001) and weakly correlated with LDL-C and HOMA-IR (both *P* < 0.05). Furthermore, the TyG index was positively correlated with the UACR (*r* = 0.19, *P* < 0.001), although no significant correlation was detected between the TyG index and baPWV. HOMA-IR was weakly correlated with the UACR (*r* = 0.11, *P* = 0.001) and baPWV (*r* = 0.08, *P* = 0.019).

### Associations of PA, the TyG index, and HOMA-IR with microalbuminuria and increased arterial stiffness (baPWV > 1400 cm/s)

Table 2 shows that microalbuminuria and increased arterial stiffness were significantly associated with a lower level of PA and a higher TyG index in both models. In contrast, the associations between HOMA-IR and microalbuminuria and increased arterial stiffness did not reach statistical significance, except for the weak association between HOMA-IR and microalbuminuria observed in Model 1.

**Table 2:** Odds ratios for microalbuminuria and increased arterial stiffness (baPWV > 1400 cm/s) according to the phase angle, TyG index, and HOMA-IR

	Microalbuminuria				Increased arterial stiffness			
	Model 1	*P*	Model 2	*P*	Model 1	*P*	Model 2	*P*
PA	0.434 (0.334 − 0.564)	< 0.001	0.410 (0.302 − 0.556)	< 0.001	0.710 (0.538 − 0.938)	0.016	0.635 (0.455 − 0.885)	0.007
T1	ref		ref		ref		ref	
T2	0.408 (0.279 − 0.597)	< 0.001	0.388 (0.255 − 0.589)	< 0.001	0.687 (0.441 − 1.070)	0.097	0.591 (0.363 − 0.963)	0.035
T3	0.313 (0.204 − 0.479)	< 0.001	0.305 (0.188 − 0.494)	< 0.001	0.565 (0.359 − 0.890)	0.014	0.467 (0.276 − 0.791)	0.005
*P* for trend	< 0.001		< 0.001		0.047		0.017	
TyG index	1.789 (1.434 − 2.232)	< 0.001	1.493 (1.158 − 1.924)	0.002	1.557 (1.207 − 2.007)	0.001	1.378 (1.037 − 1.832)	0.027
T1	ref		ref		ref		ref	
T2	1.560 (1.039 − 2.344)	0.032	1.371 (0.881 − 2.131)	0.162	1.485 (0.984 − 2.242)	0.060	1.425 (0.908 − 2.236)	0.123
T3	2.641 (1.784 − 3.911)	< 0.001	1.727 (1.103 − 2.703)	0.017	1.970 (1.289 − 3.009)	0.002	1.625 (1.013 − 2.609)	0.044
*P* for trend	< 0.001		0.057		0.007		0.116	
HOMA-IR	1.024 (1.001 − 1.048)	0.042	1.020 (0.997 − 1.043)	0.089	1.031 (0.979 − 1.086)	0.245	1.014 (0.972 − 1.058)	0.515
T1	ref		ref		ref		ref	
T2	0.808 (0.543 − 1.202)	0.292	0.657 (0.426 − 1.012)	0.057	1.465 (0.977 − 2.196)	0.065	1.395 (0.899 − 2.164)	0.137
T3	1.267 (0.870 − 1.845)	0.217	1.065 (0.681 − 1.667)	0.781	2.031 (1.327 − 3.107)	0.001	1.729 (1.066 − 2.805)	0.027
*P* for trend	0.068		0.049		0.005		0.078	

Model 1: adjusted for age and sex; Model 2: adjusted for age, sex, BMI, duration of diabetes, SBP, DBP, HbA1c, LDL, HDL, eGFR, hypertension, coronary heart disease, smoking, and drinking

We further explored the associations by categorizing PA, the TyG index and HOMA-IR into tertiles, using the first tertile as a reference. After full adjustment, the analysis revealed that the highest PA group was significantly associated with lower prevalences of both microalbuminuria and increased arterial stiffness than the lowest PA group, with ORs and 95% CIs of 0.305 (0.188 − 0.494) and 0.467 (0.276 − 0.791) and *P* trends < 0.001 and 0.017, respectively. Conversely, the highest TyG group was associated with increased prevalences of microalbuminuria and increased arterial stiffness, with ORs and 95% CIs of 1.727 (1.103 − 2.703) and 1.625 (1.013 − 2.609), respectively, but the *P* trends were not statistically significant. Consequently, PA is independently and more strongly associated with microalbuminuria and increased arterial stiffness than the TyG index and HOMA-IR are.

**Fig. 3:**
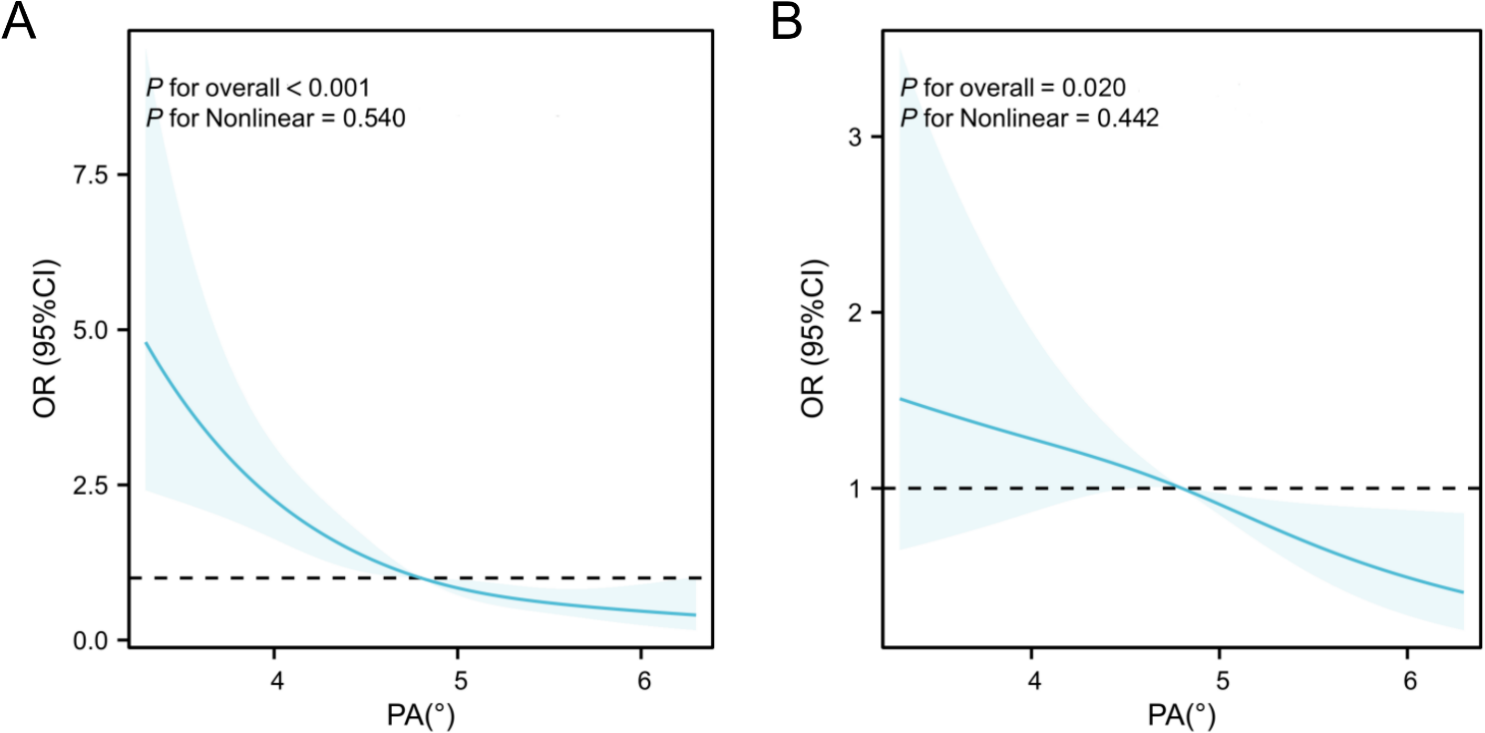
Association of the phase angle with microalbuminuria and increased arterial stiffness. Restricted cubic spline of the linear trends between the phase angle and the prevalence of microalbuminuria **(A)** and increased arterial stiffness **(B)**, adjusted for age, sex, BMI, duration of diabetes, SBP, DBP, HbA1c, LDL, HDL, eGFR, hypertension, coronary heart disease, smoking, and drinking

The RCS model demonstrated a significant negative linear dose-response relationship between PA and microalbuminuria (Fig. 3A, P for overall < 0.001), as did increased arterial stiffness (Fig. 3B, P for overall = 0.020). Additionally, the nonlinear dose-response associations of microalbuminuria and increased arterial stiffness with the TyG index are presented in Supplementary Figure S1.

### Subgroup analysis for the association of PA, the TyG Index, with microalbuminuria and increased arterial stiffness (baPWV > 1400 cm/s)

**Fig. 4:**
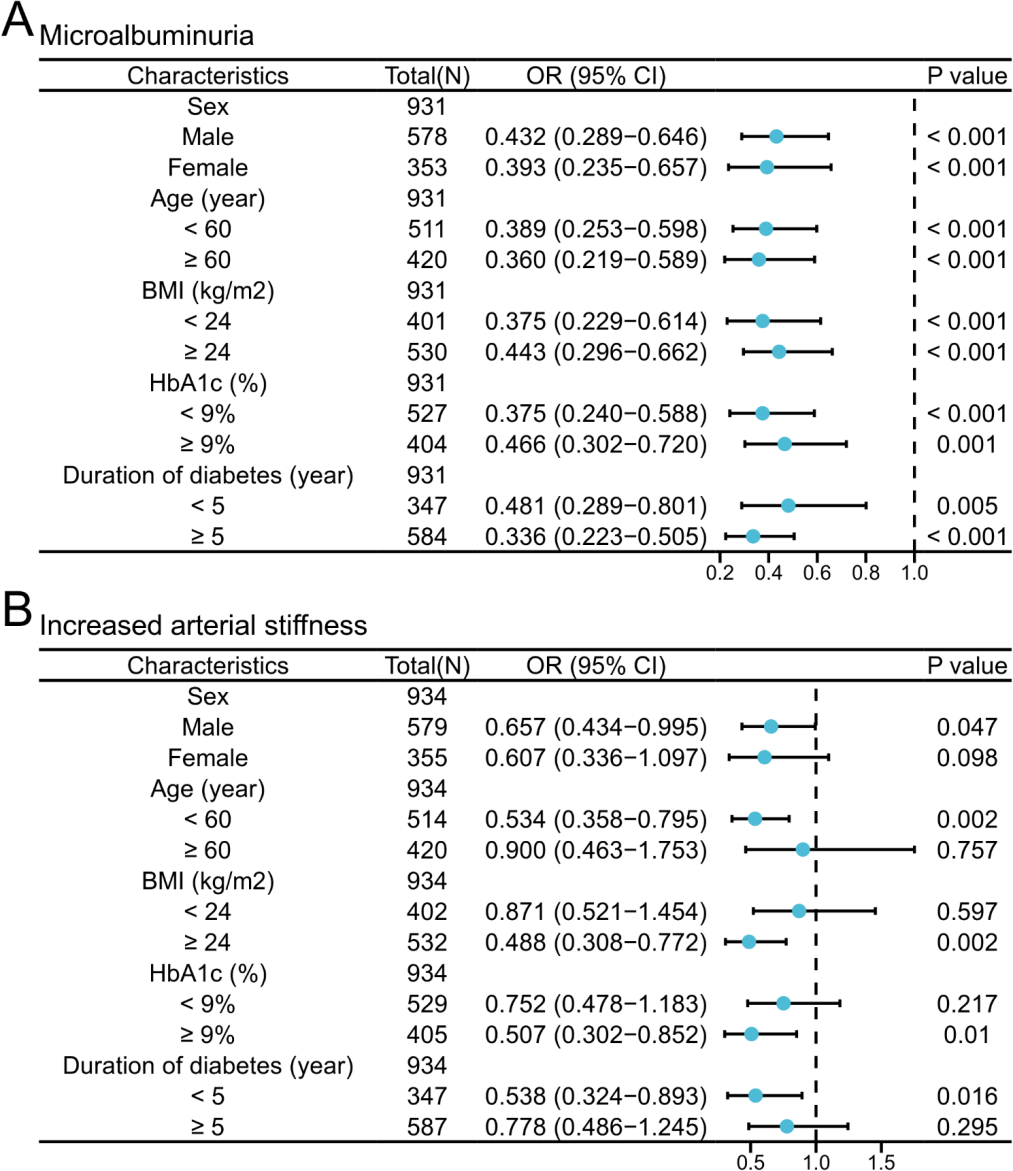
Subgroup analyses of the association of phase angle with microalbuminuria and increased arterial stiffness. Odds ratios of microalbuminuria **(A)** and increased arterial stiffness **(B)** according to phase angle stratified by sex, age, BMI, HbA1c, and duration of diabetes, adjusted for age, sex, BMI, duration of diabetes, SBP, DBP, HbA1c, LDL, HDL, eGFR, hypertension, coronary heart disease, smoking, and drinking

Furthermore, we conducted a subgroup analysis to examine the associations between PA and the prevalence of microalbuminuria and increased arterial stiffness. This analysis employed binary logistic regression models stratified by sex, age, BMI, HbA1c, and duration of diabetes. The models were fully adjusted for anthropometric, clinical, and sociodemographic variables. PA was negatively associated with microalbuminuria in all subgroups (all *P* < 0.01) (Fig. 4A) and was significantly negatively associated with increased arterial stiffness in subgroups characterized by male sex, age < 60 years, BMI ≥ 24 kg/m^2^, HbA1c ≥ 9%, and diabetes duration < 5 years (Fig. 4B). Additionally, significant positive associations between the TyG index and microalbuminuria were observed in specific subgroups, except for female subjects, individuals aged ≥ 60 years, those with HbA1c < 9%, and individuals with a duration of diabetes < 5 years. Moreover, the associations between the TyG index and increased arterial stiffness were significant and only marked by females, those aged ≥ 60 years, those with a BMI ≥ 24 kg/m^2^, and those with a duration of diabetes ≥ 5 years (Supplementary Figure S2). These findings further indicate that PA is more strongly associated with microalbuminuria and increased arterial stiffness than the TyG index is despite the relatively weak association between PA and increased arterial stiffness.

## Discussion

### Main findings

To our knowledge, this study is the first to demonstrate PA in the context of diabetic vascular complications. As a bioelectric property of the organism, PA is significantly associated with microalbuminuria and increased arterial stiffness in patients with T2D, independently of traditional risk factors such as age, sex, BMI, HbA1c, duration of diabetes, blood pressure, serum lipid levels, eGFR, smoking, drinking, and history of CHD and hypertension. Compared with the TyG index and HOMA-IR, PA exhibited stronger associations with these vascular complications. A negative linear dose-response relationship was also observed between PA and both outcomes. The negative association between PA and microalbuminuria was consistent across various subgroups, underscoring its universal applicability. However, its inverse association with increased arterial stiffness was pronounced only in specific subgroups.

### PA and microvascular damage

PA is an emerging biological parameter that indicates cellular health and has intrinsic prognostic value. There are few comparable studies designed to examine the relationship between PA and diabetic complications. Schimpfle et al. reported that T2D patients with diabetic polyneuropathy (DPN) have lower PA compared to T2D patients without DPN and healthy controls. Moreover, the PA is an investigator-independent marker for detecting DPN [27, 28]. In patients with diabetic CKD stage 5 (DMCKD5), lower PA (< 4.17°), which represents malnutrition status, was significantly associated with lower eGFR, lean tissue indices, and albumin levels, suggesting that PA could be used as a marker to reflect nutritional status in patients with DMCKD5 [29]. This is consistent with the correlation analysis in our study, showing a positive link between PA and eGFR, with the average eGFR exceeding 90.

The UACR is a sensitive indicator reflecting early renal function injury. In the present study, we found that lower PA was associated with a higher risk of microalbuminuria, even in the subgroups stratified by sex, age, BMI, HbA1c, and duration of diabetes. The strong association between PA and microalbuminuria may be attributed to its ability to reflect cellular integrity and metabolic status, which are closely linked to glomerular damage and urinary albumin excretion. A low PA level may reflect impaired cell membrane function, which is related to microvascular endothelial cell damage caused by diabetes. In previous studies, the researchers used cluster analysis by clusters of body fat and nutritional parameters, including PA, and reported that those with poor nutritional parameters were more likely to have lower eGFR and increased UACR [30], which is consistent with our study. Similar to the observations of Low et al., lower PA was associated with older age, longer duration of diabetes, higher UACR, and lower eGFR in patients with T2D. Further mechanism exploration revealed that lower PA was associated with CKD progression through MMP-2, a zinc-dependent endopeptidase family member, and promoted renal interstitial fibrosis [31].

### PA and macrovascular damage

Macrovascular diseases, including CHD, stroke, and peripheral artery disease, are among the complications of T2D and represent the leading causes of morbidity and mortality in patients with T2D [32]. Our findings align with those of Bellido et al., who reported the utility of PA in assessing systemic inflammation and metabolic disturbances [4], further expanding its application to diabetes-related vascular damage. The association between PA and arterial stiffness may be mediated by its impact on systemic inflammation and oxidative stress, contributing to vascular remodeling. In line with previous publications, low PA suggests impaired cellular function, which may be related to chronic inflammation and oxidative stress, which are central to the pathogenesis of diabetic macrovascular disease and other systemic metabolic abnormalities [33].

A higher PA reflects better cellular health or reduced cellular stress [4], whereas a lower baPWV indicates better vascular elasticity and slower pulse wave propagation [34]. It has been reported that PA is independently associated with pulse wave velocity (PWV) in peritoneal dialysis patients after adjusting for age, sex, diabetes, CRP, and eGFR [35]. Moreover, the significant correlation between nutritional indicators and PWV in peritoneal dialysis patients is unrelated to inflammation and diabetic status, suggesting that malnutrition may be one of the causes of vascular dysfunction [35]. In a diabetes-dominant cohort, PA was strongly negatively correlated with markers associated with adverse cardiovascular characteristics, including higher carotid-femoral PWV (cfPWV), which indicates its potential for early identification of vascular abnormalities in patients with diabetes [36]. These findings support the potential use of PA as an indicator for vascular health assessment in patients with diabetes or other chronic diseases. However, unlike our study and most others, one study reported that 50-kHz whole-body PA positively correlates with PWV in overweight individuals and obese individuals, although the correlation was weak [37]. We demonstrated that PA is negatively associated with increased arterial stiffness in all patients with T2D; however, a significant association occurred only in subgroups characterized by male sex, age < 60 years, BMI ≥ 24 kg/m^2^, HbA1c ≥ 9%, and diabetes duration < 5 years. These findings indicate that the relationship between PA and arterial stiffness may vary depending on the study population or specific measurement conditions. Further studies are needed to clarify these discrepancies and explore the underlying mechanisms involved.

### The better clinical application of PA in T2D vascular complications than the TyG index and HOMA-IR

The TyG index and HOMA-IR are used to assess IR and are regarded as significant contributing factors for arterial stiffness and CVD due to the production of inflammatory factors and subsequent endothelial damage, of which the former might be a better indicator [17, 18]. IR is consistently associated with elevated urinary albumin excretion, primarily as a result of alterations in renal endothelial function and hemodynamics [38, 39]. Among community‑dwelling elderly individuals, an elevated TyG index was significantly associated with a greater risk of cfPWV > 10 m/s, baPWV > 1800 cm/s, microalbuminuria, and CKD, which indicated that the TyG index could be a predictor of arterial stiffness and nephric microvascular damage [19]. Chiu et al. reported a high TyG index and a high risk of microalbuminuria and cerebrovascular disease in a study cohort of patients with T2D but not CHD [20]. Yan et al. reported that an elevated TyG index and HOMA-IR were associated with a greater risk of diabetic kidney disease (DKD) (microalbuminuria or eGFR < 60) in patients with T2D. However, receiver operating characteristic curves revealed that the HOMA-IR was better than the TyG index at predicting the risk of DKD [40]. In our study, after adjusting for confounders, the TyG index was significantly associated with microalbuminuria and increased arterial stiffness, but the associations were weaker than those of PA. Subgroups analysis further revealed stronger associations between PA and these complications compared to the TyG index. Additionally, the associations between HOMA-IR and microalbuminuria and increased arterial stiffness did not reach statistical significance in our study. These findings collectively suggest that PA is independently and has a stronger association with microvascular and macrovascular complications than the TyG index and HOMA-IR. Although the mechanisms have not yet been fully elucidated, they may be attributed to PA, which comprehensively covers inflammation, oxidative stress, and cellular damage associated with vascular injury.

### Limitations

This study has certain limitations. The study has a cross-sectional design, so a causal relationship between PA and microalbuminuria or increased baPWV cannot be established. Future longitudinal studies are needed to validate whether changes in PA over time can predict the onset or progression of these conditions. Although this study adjusted for several potential confounders, residual confounding may still exist. Unmeasured factors, such as diet, physical activity, or medication use, may affect the results.

## Conclusion

Compared with the TyG index and HOMA-IR, the PA is independently and more strongly associated with micro- and macrovascular complications in patients with T2D. PA is particularly associated with microvascular complications, but its association with macrovascular complications is relatively weak.

Considering that PA is a noninvasive and convenient indicator, it may be more suitable than the TyG index and HOMA-IR for the early assessment of vascular status in patients with T2D, particularly in the absence of apparent clinical symptoms. Patients with lower PA may require more intensive vascular health management strategies to prevent or delay the onset of diabetes-related micro- and macrovascular complications.

## Electronic supplementary material

Below is the link to the electronic supplementary material.

Supplementary Material 1

## Data Availability

The datasets generated during and/or analyzed during the current study are not publicly available but are available from the corresponding author upon reasonable request.

## Abbreviations

baPWV: brachial-ankle pulse wave velocity
BIA: Bioelectrical impedance analysis
BMI: Body mass index
cfPWV: carotid-femoral pulse wave velocity
CHD: Coronary heart disease
CI: Confidence interval
CKD: Chronic kidney disease
CVD: Cardiovascular disease
DBP: Diastolic blood pressure
DKD: Diabetic kidney disease
eGFR: estimated glomerular filtration rate
FIB-4: Fibrosis-4 score
FINS: Fasting serum insulin
FPG: Fasting plasma glucose
HbA1c: Glycated hemoglobin
HDL-C: High-density lipoprotein cholesterol
HOMA-IR: Homeostasis model assessment for insulin resistance
LDL-C: Low-density lipoprotein cholesterol
OR: Odds ratio
PA: Phase angle
PWV: Pulse wave velocity
SBP: Systolic blood pressure
SFA: Subcutaneous fat area
T2D: Type 2 diabetes
TC: Total cholesterol
TyG: Triglyceride‒glucose index
UACR: Urinary albumin-to-creatinine ratio
VFA: Visceral fat area
WC: Waist circumference
2h-PG: 2-hour postprandial blood glucose

## Declarations

### Clinical trial information

This study was approved by the Ethics Committee of Jiangsu Provincial People’s Hospital (2021-SR-298).
