# Supplementary material for: Stronger associations of the phase angle than the TyG index with micro- and macrovascular complications in patients with type 2 diabetes
Source: Lipids Health Dis. 2025 Apr 1;24:125. doi: 10.1186/s12944-025-02534-5 (PMC11959766; doi:10.1186/s12944-025-02534-5)
Supplement: Supplementary file 1 — Supplementary Material 1 [file 12944_2025_2534_MOESM1_ESM.docx]

**Supplementary materials**

**
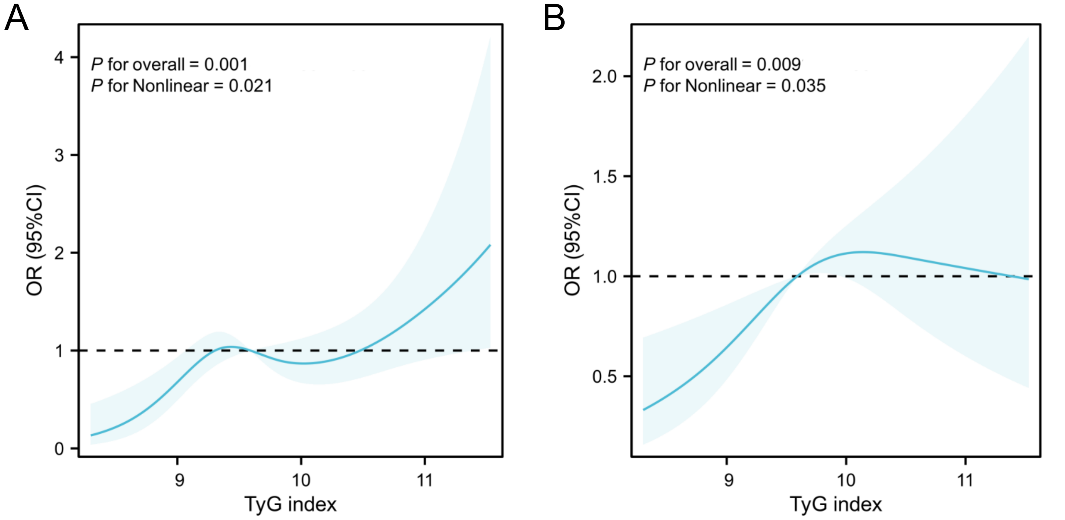
**

**Fig. S1 Association of the TyG index with microalbuminuria and increased arterial stiffness.**

Restricted cubic spline of the non-linear trends between the TyG index and the prevalence of microalbuminuria (A) and increased arterial stiffness (B), adjusted for age, sex, BMI, duration of diabetes, SBP, DBP, HbA1c, LDL, HDL, eGFR, hypertension, coronary heart disease, smoking, and drinking.


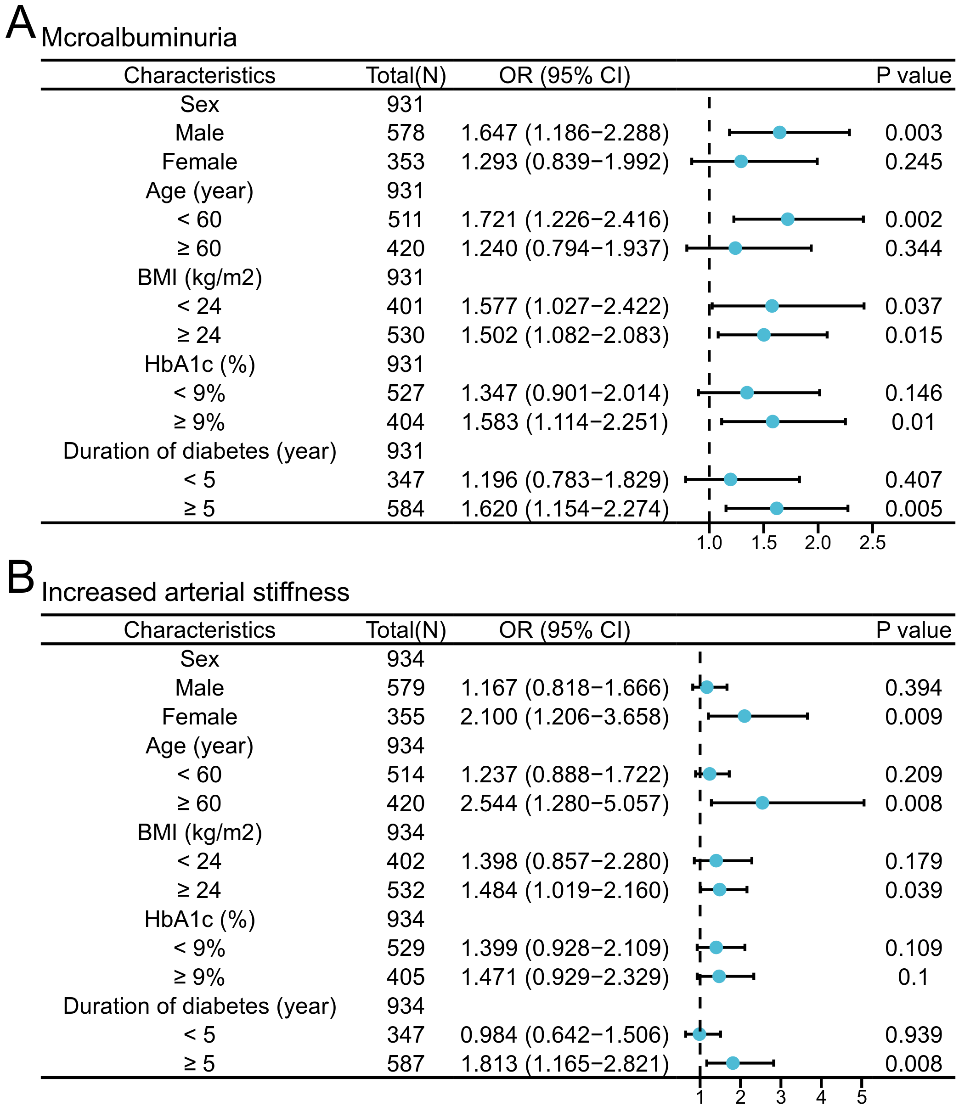


**Fig. S2 Subgroup analyses of the association of the TyG index with microalbuminuria and increased arterial stiffness**

Odds ratios of microalbuminuria (A) and increased arterial stiffness (B) according to the TyG index stratified by sex, age, BMI, HbA1c, and duration of diabetes, adjusted for age, sex, BMI, duration of diabetes, SBP, DBP, HbA1c, LDL, HDL, eGFR, hypertension, coronary heart disease**,** smoking, and drinking.
